# Impact of motorboats on fish embryos depends on engine type

**DOI:** 10.1093/conphys/coy014

**Published:** 2018-03-13

**Authors:** Sofia Jain-Schlaepfer, Eric Fakan, Jodie L Rummer, Stephen D Simpson, Mark I McCormick

**Affiliations:** 1 ARC Centre of Excellence for Coral Reef Studies, and College of Science and Engineering, James Cook University, Townsville, Queensland 4811, Australia; 2 Biosciences, College of Life and Environmental Sciences, University of Exeter, Geoffrey Pope, Stocker Road, Exeter EX4 4QD, UK

**Keywords:** Anthropogenic noise, boats, embryo, fishes, pollution, stress

## Abstract

Human generated noise is changing the natural underwater soundscapes worldwide. The most pervasive sources of underwater anthropogenic noise are motorboats, which have been found to negatively affect several aspects of fish biology. However, few studies have examined the effects of noise on early life stages, especially the embryonic stage, despite embryo health being critical to larval survival and recruitment. Here, we used a novel setup to monitor heart rates of embryos from the staghorn damselfish (*Amblyglyphidodon curacao*) in shallow reef conditions, allowing us to examine the effects of *in situ* boat noise in context with real-world exposure. We found that the heart rate of embryos increased in the presence of boat noise, which can be associated with the stress response. Additionally, we found 2-stroke outboard-powered boats had more than twice the effect on embryo heart rates than did 4-stroke powered boats, showing an increase in mean individual heart rate of 1.9% and 4.6%, respectively. To our knowledge this is the first evidence suggesting boat noise elicits a stress response in fish embryo and highlights the need to explore the ecological ramifications of boat noise stress during the embryo stage. Also, knowing the response of marine organisms caused by the sound emissions of particular engine types provides an important tool for reef managers to mitigate noise pollution.

## Teaser

Boat noise is becoming increasingly common and is now recognized as an environmental pollutant (Slabbekoorn *et al.*, 2010). We show, for the first time that boat noise elicits a stress response in fish embryos, and that their stress response is greater upon exposure to 2-stroke engines than 4-stroke engines.

## Introduction

Human generated noise is changing natural soundscapes worldwide. Boat noise is the most prevalent source of underwater anthropogenic noise and is becoming recognized in international legislation as a prevalent anthropogenic pollutant that is increasing (Slabbekoorn *et al.*, 2010; International Maritime Organization, 2011; Badino *et al.*, 2012; Borsani *et al.*, 2015). While boat noise has been found to have a variety of biological impacts on a broad range of taxa (Rolland *et al.*, 2012; Nedelec *et al.*, 2014; Simpson *et al.*, 2016), data are insufficient to provide the evidence needed to inform policy geared toward mitigating biological and environmental impacts. Current boat noise regulations are developed based on assessments of airborne emissions affecting comfort of onboard living conditions or that of inhabitants near ports, but not the impacts of noise on aquatic life (Badino *et al.*, 2012). Successful mitigation likely depends on altering boat noise production rather than decreasing boat prevalence, because boat numbers continue to increase. Yet, to our knowledge, no studies have examined the responses of aquatic organisms to noise from different types of boat engines.

The early life stages of marine organisms can be particularly susceptible to environmental perturbations, especially at key development stages when sensitivities are high (Mager *et al.*, 2017). While most research that documents the importance of the early life history to population dynamics focuses on the larval phase (Peck *et al.*, 2012), it is of course preceded in most species by an egg phase whose sole purpose is development driven and fuelled by maternally provisioned endogenous yolk reserves. Because of the small size and rapid development, embryos are particularly sensitive to disruption by environmental perturbations (e.g. temperature shock, pollutants) with carryover effects for neural, sensory, muscular and morphological development (Roussel, 2007; McCormick and Gagliano, 2010), that may flow on to effect growth and survival (Gagliano *et al.*, 2007).

Here we investigate a coral reef fish species during the vulnerable embryonic life stage; a life stage identified as a research priority in relation to anthropogenic noise effects by the European Commission in the Marine Strategy Framework Directive (Borsani *et al.*, 2015). Boat noise has been shown to affect many biological processes in fish including parental care (Nedelec *et al.*, 2017), navigation (Holles *et al.*, 2013), foraging (Voellmy *et al.*, 2014) and survival under a predator threat (Simpson *et al.*, 2016). However, to our knowledge only a single study has examined effects of noise on fish at the embryonic life stage (Bruintjes and Radford, 2014), despite evidence suggesting that fishes begin to respond to sound during embryonic development (Simpson *et al.*, 2005) and that embryo health is important to larval growth and cohort survival (Bailey and Houde, 1989; McCormick and Nechaev, 2002; Simpson *et al.*, 2005). The present study represents a significant advancement on Bruintjes and Radford’s study by manipulating the embryos’ acoustic environment in the field using real boats and by considering both the pressure and particle motion conditions during experimental exposures. Additionally, we compare effects of 2-stroke outboard engines to quieter 4-stroke engines.

We use heart rate as an indicator of the stress response in fish embryos. Heart rate is a reliable indicator of stress and has been frequently employed as an indicator in other studies (Nimon *et al.*, 1996; Bunt *et al.*, 2004; von Borell *et al.*, 2007; Graham and Cooke, 2008; Atherton and McCormick, 2015). Heart rate increases (β-adrenoreceptor-mediated) directly in response to stressors caused by the stimulation of the hypothalmic-sympathetic-chromaffin-cell axis and the production of catecholamines (Barton, 2002; Bagatto, 2005). Therefore, heart rate provides a logistically feasible indicator of stress response, suggesting increased energy mobilization and use in fish embryo.

## Materials and methods

### Study species and collection

The staghorn damselfish (*Amblyglyphidodon curacao*) is an omnivorous damselfish that forms pairs during the breeding season when males make nests on vertical projections of dead substrate (Goulet, 1995). Eggs are laid in a monolayer and are defended from predators, principally by the male. At Lizard Island on the northern Great Barrier Reef, Australia (14°41′S, 145°27′E), during summer sea temperatures of approximately 28°C, embryos hatch 5 days post fertilization. The sagittal otoliths that form the basis of the acousticolateralis system form during embryogenesis, and it is likely that these embryos have a functioning acoustic system prior to hatching (Simpson *et al.*, 2005). As embryo do not have a gas filled swim bladder, sound detection is likely driven by particle motion auditorily and via neuromast cells (Sarrazin *et al.*, 2010). For the purposes of this paper, ‘hearing’ is used to describe the general detection of sound, via either mechanism.

Four-day-old *A. curacao* embryos were collected from the reefs around Lizard Island from 12 clutches and 9 different nesting sites/fathers between 21 and 29 October 2016. In order to collect and age the embryos, sheets of clear plastic were wrapped around dead coral branches at breeding sites and monitored daily for egg deposition. Plastic sheets were collected 4 days after egg deposition and placed into a seawater filled 9 L plastic bag, which was then placed into a seawater filled polystyrene box (to reduce noise disruption and temperature change) and driven slowly by boat (with a quiet 4-stroke engine) to a nearby beach (see Supplementary material Fig. S1 for an analysis of acoustic exposure during transport). Eggs were then kept in the plastic bags within the polystyrene box in the shade on the beach, isolated from any further boat noise, until their experimental treatment (less than 4 h later). Seawater was replaced in the plastic bags and box every 30 min, and water temperature was kept within 1°C of local sea temperature.

### Acoustic stimuli

Three different acoustic stimuli were used in experimental treatments: ambient conditions (with background biophonic noise produced by fishes and invertebrates resident on patch reefs within the bay, but without any boats operating in the area), 2-stroke powered boats, and 4-stroke powered boats. Boat stimuli consisted of boats driven at 0–35 km/h at 10–200 m from the experimental setup. Seven boats were used in total; four aluminium-hulled 5m long boats with 30 hp Suzuki 2-stroke outboard engines (model DT30) and three boats of the same design but with 30 hp 4-stroke outboard engines (model DF30A).

In order to characterize the differences in acoustic conditions in the experiment, three recordings of acoustic pressure and particle motion conditions were made for each of the treatments, where a different boat was used in each of the boat noise recordings. Recordings were made at the location of the experimental trials, 1 m above the ocean bottom, from a kayak in 2–5 m water. Acoustic-pressure recordings were taken using an omnidirectional hydrophone (HiTech HTI-96-MIN with inbuilt preamplifier, manufacturer-calibrated sensitivity −164.3 dB re 1 V/μPa; frequency range 0.02–30 kHz; calibrated by manufacturers; High Tech Inc., Gulfport MS). Particle motion recordings were taken simultaneously using a triaxial accelerometer (M20L; sensitivity following a curve over the frequency range 0–2 kHz; calibrated by manufacturers; Geospectrum Technologies, Dartmouth, Canada). Both the accelerometer and hydrophone were connected to a digital 8-track recorder (F8 field recorder, sampling rate 48 kHz, Zoom Corporation, Tokyo, Japan). Using the same recording equipment, a recording was made in the polystyrene container of seawater on a boat to quantify the acoustic conditions to which embryos were exposed during transport (Fig. S2). Calibration parameters for the recording levels used were determined by recording a pure sine wave signal from a function generator, with the voltage measured using an in-line oscilloscope. Sound files were cropped in Audacity 2.1.2 (http://www.audacityteam.org), and acoustic analyses were calculated using PaPAM 0.872 (Nedelec *et al.*, 2016) in Matlab Compiler Runtime 8.3 (https://au.mathworks.com).

**Figure 1:**
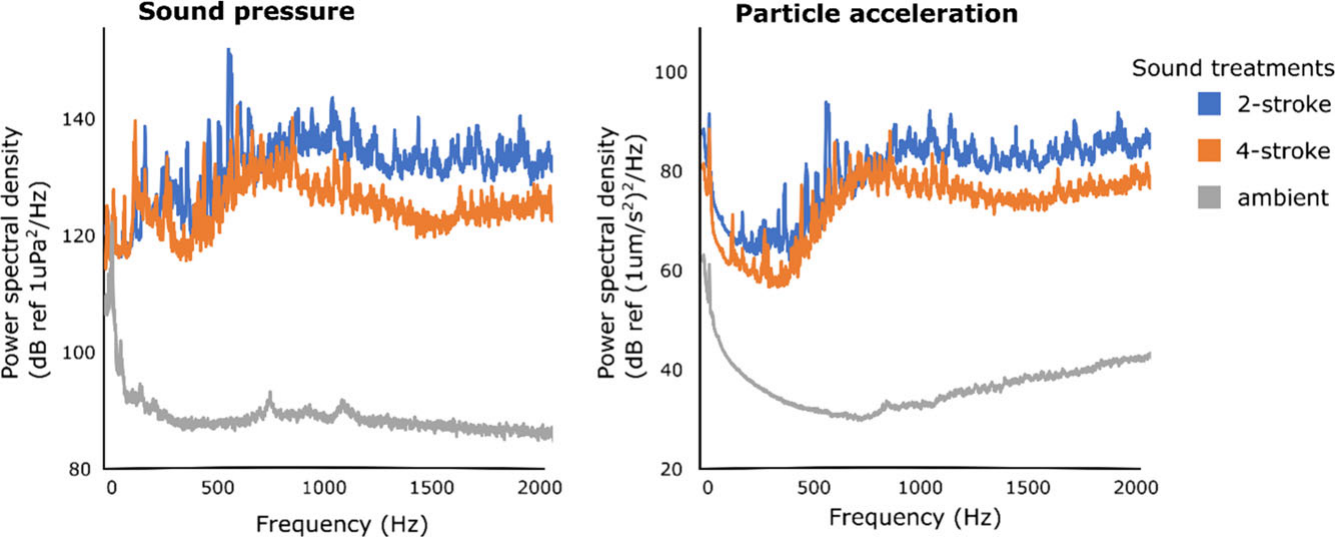
Power spectral density of the sound treatments to which *Amblyglyphidodon curacao* embryos were exposed: 2-stroke powered boat noise, 4-stroke powered boat noise and natural ambient conditions. Spectral content is shown in sound pressure (left) and particle acceleration (right). Analyses were conducted in paPAM using one minute tracks that combined three separate recordings of each treatment to give the average sound profile of the three recordings. For boat tracks, each of the three recordings used in a track were from a different boat to account for variability between boats with the same engine type.

The root mean square of the power spectral density (PSD) was used to characterize the acoustic treatments. The PSD describes the acoustic power across frequencies, while the root mean square (RMS) of the PSD provides an average across frequencies (Merchant *et al.*, 2015). The RMS PSD of each treatment (ambient, 2-stroke and 4-stroke) was calculated for 1 min tracks, where three passes of different boats of the same treatment were spliced together (to get an average between soundscape replicates), or three ambient tracks were spliced together in the case of the ambient treatment. The sound exposure level (SEL) within the estimated hearing range of the embryos (400–1200 Hz; Table 1), which describes the cumulative sound energy over time (Merchant *et al.*, 2015), was calculated for 4 min tracks of each of the three soundscape replicates of each of the treatments, and then an average was taken for each treatment. For boat treatments, every sample consisted of a different boat passing the recording equipment 11 times. Consistency analysis, which indicates the percentage of time that the amplitude of sound is greater than a given threshold (Nedelec *et al.*, 2016), was also calculated for these 4 min tracks at a threshold of 100 dB and 110 dB (at 400–1200 Hz) for particle motion and pressure, respectively. These thresholds are the best estimate of the embryos hearing thresholds based on previous studies with pomacentrid fishes (Table 1). Consistency was then multiplied by SELs to give an estimate of the cumulative sound energy that embryos were exposed to for each treatment (Fig. 1).

**Table 1:** Approximate hearing thresholds found in other studies on pomacentrid fishes

Reference	Species	Life stage	Hearing range (Hz)	Pressure threshold range (dB re 1 μPa)	Acceleration threshold range (dB re 1 μm/s^2^)
Wysocki *et al.* (2009)	*Chromis chromis*	Adult	100–500	100–110	65–75
Wright *et al.* (2011)	*Pomacentrus nagasakiensis, Pomacentrus amboinensis*	Settlement stage larvae	100–2000	120–140	95–105
Kenyon (1996)	*Pomacentrus variablis*	Post-settlement juvenile	300–1200	At 13mm: 120–140 At 20mm: 110–130	
Egner (2004)	*Abudefduf saxatilis*	Post-settlement juveniles	100–1200	110–150	
Simpson *et al.* (2005)	*Amphiprion ephippium*	Embryo	At 3 days: 400–700 At 9 days: 400–1200	At day 3: 140–150 At day 9: 100–140	

### Experimental design

**Figure 2:**
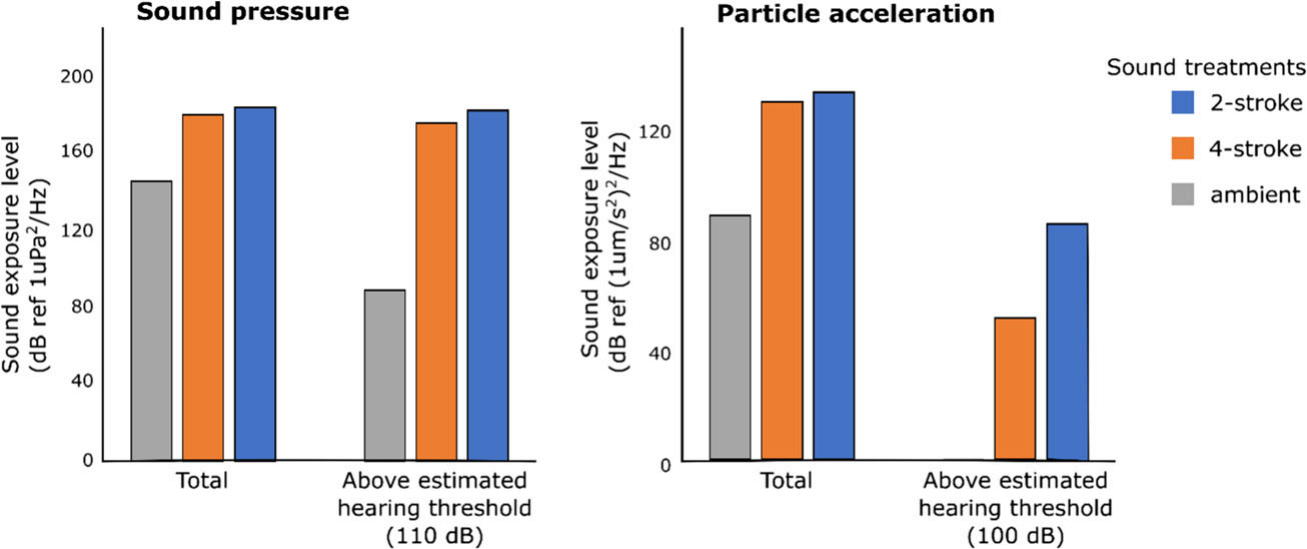
Sound exposure levels (SELs) are the cumulative sound energy at 400–1200 Hz (the estimated hearing range of *Amblyglyphidodon curacao* embryo) over 4 min. The SELs are shown in sound pressure (left) and particle acceleration (right) for each of three sound treatments: a 2-stroke powered boat, a 4-stroke powered boat noise, and natural ambient conditions. Total SELs as well as the SELs above the estimated hearing thresholds of *A. curacao* embryos (110 dB sound pressure; 100 dB particle acceleration) were calculated. Analyses were conducted using paPAM on three 4 min tracks for each treatment. The mean SEL of each treatment is represented in the graph. For boat tracks, each of the three recordings used in computing the average SEL were from different boats to account for variability between boats with the same engine type.

Heart rate was measured using a recording apparatus located on a shallow (2–5 m) sandy bottom site, adjacent to a reef (25 metres), in front of Lizard Island Research Station (14° 40′S, 145° 28′E), Great Barrier Reef, Australia. The apparatus consisted of an Olympus Stylus T-4 camera with an i-Das UCL-02 lens (125 mm/+8 macro lens), a Perspex stage in front of the lens, and a dive torch. To film the embryos, a strip of plastic sheet onto which embryos had been laid was attached to the stage and illuminated from behind by the torch (see Supplementary material Fig. S2 for photograph). Before each trial, a strip of the plastic sheet containing the egg clutch was cut off and taken by a snorkeler to the video apparatus. The camera was then focused on one to four individual embryos with visible heartbeats. Following 15 min of habituation in ambient conditions (natural ambient sound in the absence of boats), heartbeats were recorded using the camera for a further 2 min under pre-treatment, ambient conditions, followed by 4 min of one of three randomly selected acoustic stimuli treatments (ambient, 2-stroke, 4-stroke, Fig. 2).

### Analyses

Heartbeats were counted in 20 s intervals, blind to treatment. To determine the time at which embryos were affected by boat noise, heart rate was initially plotted over time for embryos exposed to ambient conditions and those exposed to 2-stroke boat noise during treatment (see Supplementary material Fig. 3). These results suggested that it takes 140 s for embryos’ hearts to reach their full response to boat noise. Therefore, heart rate measurements taken in the 2 min following the first 140 s of boat noise were averaged within individuals to represent heart rate during treatments, and the heart rate measurement taken during the 2 min of pre-treatment, ambient conditions were averaged within individuals to represent heart rate during pre-treatment.

**Figure 3:**
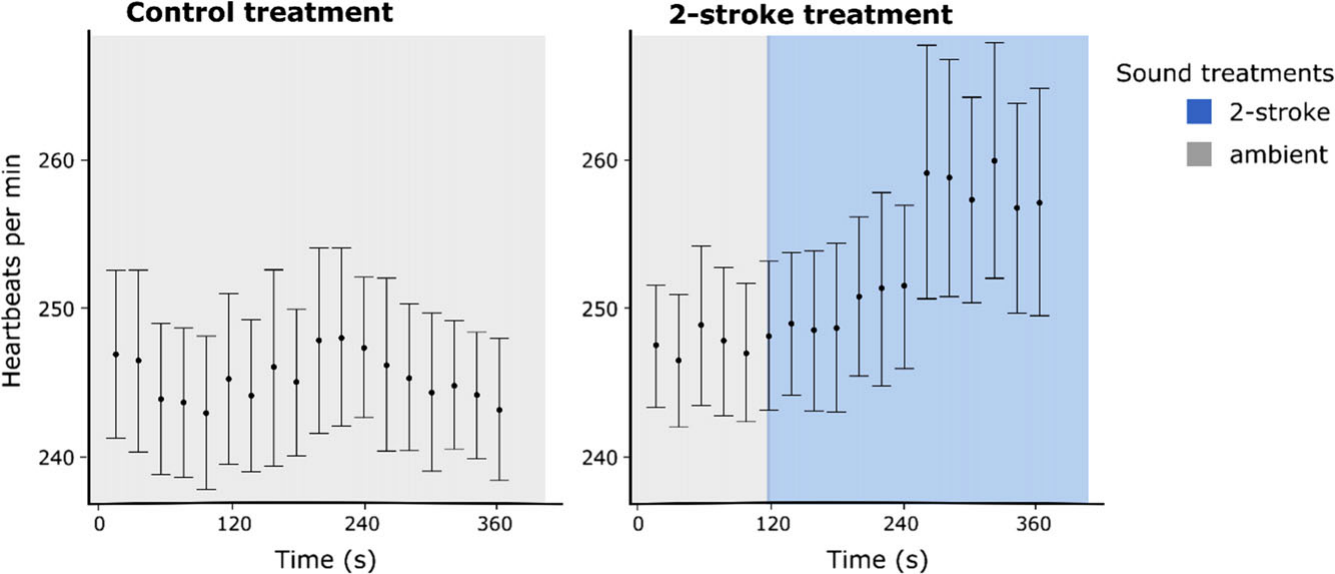
Heart rate of 4 day old *Amblyglyphidodon curacao* embryos, following 15 min of habituation in the recording apparatus. Embryos were exposed to either ambient conditions for 360 s (left; sample size 13) or 120 s in ambient conditions followed by 240 s of 2-stroke powered boat noise driving at 0–35 km/h at 10–200 m from embryos (right; sample size 18). The full heart rate response to boat noise appears to occur 140 s after initiation of exposure to boat noise.

A linear mixed effects split-plot model was fitted to the data using maximum likelihood and implemented using the ‘lmer’ function in the ‘lme4’ R package (Bates *et al.*, 2015). Treatment was included as a between individuals fixed effect, and time (pre-treatment/treatment) was included as a within individuals fixed effect, thereby incorporating the repeated measures element of the design into the analysis. A fixed treatment–time interaction was included to determine whether changes in heart rates within individuals differed with treatment. Individual and clutch were added as random factors without interactions. The assumption of normality was met, and the response variable (heart rate) was square root transformed to meet the assumption of homogeneity of variance. Within-group correlation structure did not improve the model and thus was not incorporated (Logan, 2010). The ‘lsmeans’ function in the ‘lsmeans’ package was used post-hoc to identify where differences among means occurred (Lenth, 2016).

## Results

### Acoustic analysis

In general, boats with 2-stroke engines generated more noise than boats 4-stroke engines. The RMS acoustic pressure (PSD) generated by boats within the estimated hearing range of *A. curacao* embryos (400–2000 Hz, Table 1) was 125 dB re μPa^2^Hz^−1^ for 4-stroke engines and 132 dB ref μPa^2^Hz^−1^ for 2-stroke engines (Fig. 2). Within this same frequency range the average particle acceleration was 77 dB re μms^−2^Hz^−1^ for 4-stroke engines and 84 dB re μms^−2^Hz^−1^ for 2-stroke engines. The cumulative sound energy over 4 min (SEL) above an estimated hearing threshold for *A. curacao* embryos of 110 dB re μPa^2^Hz^−1^ and within their estimated hearing range (400–2000 Hz) was 89 (ambient conditions), 175 (4-stroke engines) and 181 dB re μPa^2^s^−2^Hz^−1^ (2-stroke engines; Fig. 1). The SEL above an estimated hearing threshold of 100 dB re μms^−2^Hz^−1^ in terms of particle motion showed an even greater difference between 4-stroke and 2-stroke engines, averaging at 51 and 86 dB re μms^−2^Hz^−1^, respectively, while for ambient conditions gave an SEL of 0.006 dB re μms^−2^Hz^−1^.

### Effect of boat noise

**Figure 4:**
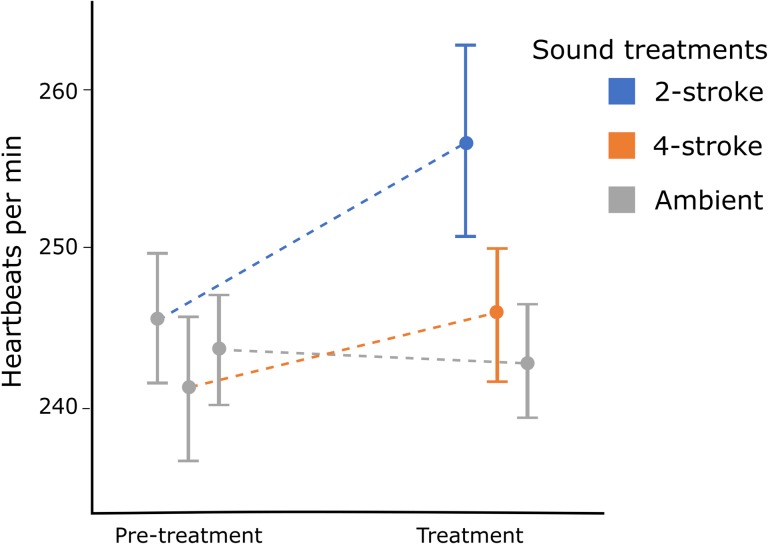
: Change in heart rate of *Amblyglyphidodon curacao* embryos from pre-treatment conditions (ambient) to treatment conditions (ambient, 2-stroke engine or 4-stroke engine), where heart rate was negated for the first 140 s of the treatment conditions to allow the response to be reached. Both 2-stroke and 4-stroke treatments involved a boat driving at 0–35 km/h at 10–200 m from embryos. The graph displays inter-individual means, and bars represent 95% confidence intervals.

When compared to embryos under ambient conditions, heart rate of *A. curacao* embryos significantly increased during exposure to boat noise, as demonstrated by a significant treatment–time (time = pre-treatment or treatment) interaction (F_2,20_ = 21.0, *P* < 0.001; Fig. 4). Embryos that were exposed only to ambient conditions did not show a significant change in heart rate between pre-treatment and treatment periods (t_66_ = 0.5, *P* = 0.6). Embryos exposed to 2-stroke engine noise showed a statistically significant mean increase in heart rate of 4.6% ± 3.5 above that under ambient conditions (t_66_ = −8.6, *P* < 0.001). The effect of 4-stroke engine noise was less than half of the effect of 2-stroke engine noise, showing a mean increase in heart rate of 1.9 % ± 1.7 from ambient conditions (t_66_ = −3.7, *P* < 0.001).

## Discussion

We found increased heart rate in *A. curacao* embryos in response to boat passage. Increased heart rate indicates the initiation of an adrenergic stress response, which is ultimately responsible for activating metabolic pathways and the mobilization of energy substrates to deal with perceived challenges (Armstrong, 1986; Lucas, 1994; Barton, 2002). In the case of boat noise, the perceived challenge is not a threat, so the energy expenditure associated with the stress could be detrimental to the embryos by depleting energy that could have otherwise been allocated to fitness promoting processes. However, from the data collected in this study, we cannot say whether the energetic cost of the stress induced by boat noise is sufficiently large to have impacts on body condition and fitness (Frid and Dill, 2002). If boat noise induced stress significantly depletes embryonic energy reserves, it may affect subsequent recruitment to coral reefs. Growth until feeding in the post-yolk sac larval stage is dependent on the available yolk sac energy reserves (McCormick and Nechaev, 2002). Thus, depletion of the endogenous embryonic energy reserves can reduce larval growth. Studies have found that larger and faster growing larvae have higher survival, which is related to larger larvae having increased ability to compete for food, resist starvation, and avoid predation (Bailey and Houde, 1989; Sogard, 1997; Jenkins and King, 2006; Peck *et al.*, 2012). Furthermore, in many populations, a strong link has been found between larval abundance and recruitment (Cushing, 1990; Leggett and Deblois, 1994; Karjalainen *et al.*, 2000). Thus, the depletion of energy reserves associated with boat noise induced stress may affect young-of-year recruitment by reducing growth. Further experiments are required to quantify the magnitude of these energetic costs associated with boat noise induced stress and determine whether these costs affect recruitment by affecting embryo survival or causing carryover effects to later life stages (McCormick and Gagliano, 2010).

The embryos heart rates increased by 1.9% and 4.9% on average with the passage of 2-stroke and 4-stroke powered boats, respectively. Any additional stress caused by the experimental procedure likely make these estimates more conservative, as they would decrease the ability of the embryo to respond to other stressors. An increase in heart rate of 4.9% with the passage of 2-stroke powered boats may indicate a considerably severe stress response when compared to increases in heart rate associated with conspecific alarm odours found in other fish species. A study on another pomacendrid (*Amphiprion melanopus*) found that embryos responded to conspecific alarm odours, arguably the most stressful cues that could be perceived, with an average increase in heart rate of 6.6% and 12.2% on Days 6 and 7 of development over an 8 day development period (Atherton and McCormick, 2015). On Day 4 of development, *Melanotaenia duboulayi* showed a 8.9% increase in heart rate in response to conspecific alarm odour (Oulton *et al.*, 2013). In a detailed experimental study of the affect of cortisol on developmental rhythms during embryogenesis, McCormick & Nechaev (2002) found that the experimental elevation of cortisol resulted in a 4–14% increase in heart rate, and that magnitude of increase was dependent upon developmental stage. Overall these changes were enough to alter the size of larvae at hatching such that larvae with higher heart rates were smaller in size. These findings were further supported by a study that looked at the interrelationships between egg, embryo and larval characteristics at the individual level (Gagliano and McCormick, 2009), suggesting that perturbations within the embryonic stage can have strong carryover effects into future life stages. Therefore, it is possible that the increases in heart rate observed in our study in the presence of boat noise may indicate a stress response that could have carryover effects to future life stages.

Our finding that *A. curacao* embryos exhibit a stress response when exposed to motorboat noise contributes to the growing body of evidence that vessel noise can have detrimental effects on fishes. At juvenile and adult life stages, several other studies have found boat noise to instigate a physiological stress response (Spiga *et al.*, 2012; Nichols, 2014; Simpson *et al.*, 2016) and behavioural changes (Holles *et al.*, 2013; Voellmy *et al.*, 2014) in fishes. At the embryonic life stage, the few studies related to boat noise show variability in the sensitivity of embryos to boat noise. The playback of chronic boat noise was not found to effect growth and survival of embryonic cichlids (*Neolamprologus pulcher*) in the laboratory (Bruintjes and Radford, 2014). In the marine mollusc *Stylocheilus striatus*, chronic boat noise playback decreased embryonic survival by 21% and by a further 22% upon hatching (Nedelec *et al.*, 2014). Differences among species in their tolerance and reaction to anthropogenic noise may be expected from differences in the development of hearing systems and their sensitivities (Wright *et al.*, 2011); a topic that remains unexplored for most species of fishes and invertebrates.

Our study is the first to assess the effects of *in situ* boat noise on embryonic fish. Using real boat noise in a field setting is an important advancement because sound is altered through its replication by speakers and by resonance, reflection, and differential absorption within a tank environment (see Rogers *et al.*, 2016 for a discussion of tank acoustics and drawbacks). Additionally, while particle motion and sound pressure components of sound have a direct relationship in the far field (fish would often experience sound in the far field in their natural environment), they do not when the sound source is in close proximity (such as a speaker in a tank). Many fishes hear both particle motion and sound pressure components of sound, and when a fish responds to sound, it is often uncertain to which component the fish is responding. Thus, it is difficult to adjust sound exposure levels in a tank experiment to the levels fish would experience in their natural environment, and therefore, it is more informative to conduct aquatic noise pollution studies in the field. It is an important advancement to find evidence that embryonic fish can display a stress response to boat noise, suggesting that it is important for future studies to examine the consequences of this stress response and whether effects carryover to later life stages. An examination of the capacity to habituate to chronic exposure would be another research direction and for future studies. However, there are potentially many confounding factors in a long term field experiment (e.g. effects on parental care, nest predators, etc.); so, our current finding that boat noise elicits an acute stress response in forms an important foundation for future work.

Another important advancement of our study is that we found the effect of boat noise on embryos differed with source of the acoustic disturbance (i.e. engine type). We found the effect of 2-stroke powered boats on embryo heart rates to be more than twice that of 4-stroke powered boats. When comparing the acoustic signatures of the two engine types, only a small difference was found in PSD, the most common metric used in noise pollution studies (Fig. 2). We suggest that measuring the total SEL above the sound pressure and particle motion hearing threshold and within the hearing range of the species and life stage may be a more appropriate metric for determining effects of noise pollution, as it is more indicative of what the organism may actually experience. There is a marked difference between 2-stroke and 4-stroke engines in the SEL produced above the estimated particle motion hearing threshold and within the hearing range of *A. curacao* embryos, which may account for the differences in heart rate responses found between the two engine types.

It is currently unclear whether the relatively small but significant changes in heart rate caused by boat noise are ecologically relevant and have repercussions for subsequent early life history dynamics. Our study lays a strong methodological foundation for further studies that will examine the potential for habituation to boat noise by embryos and the relative importance of carryover effects to later life stages. It is only by examining how noise perturbations affect all major life stages that the importance of windows of developmental sensitivity (*sensu*Fawcett and Frankenhuis, 2015) and carryover effects can be integrated into our understanding of how environmental perturbations such as noise affect the dynamics of marine organisms. Knowing whether different types of engines produce different magnitudes of disturbance is important as it gives aquatic resource managers an effective tool with which to mitigate the impacts of noise through restrictions on maximum sound outputs.

## Supplementary Material

Supplementary Data
