# Supplementary material for: Impact of motorboats on fish embryos depends on engine type
Source: Conserv Physiol. 2018 Mar 13;6(1):coy014. doi: 10.1093/conphys/coy014 (PMC5865524; doi:10.1093/conphys/coy014)
Supplement: Supplementary Data [file coy014supplimentarymaterialconsphys.docx]

# Supplementary Material


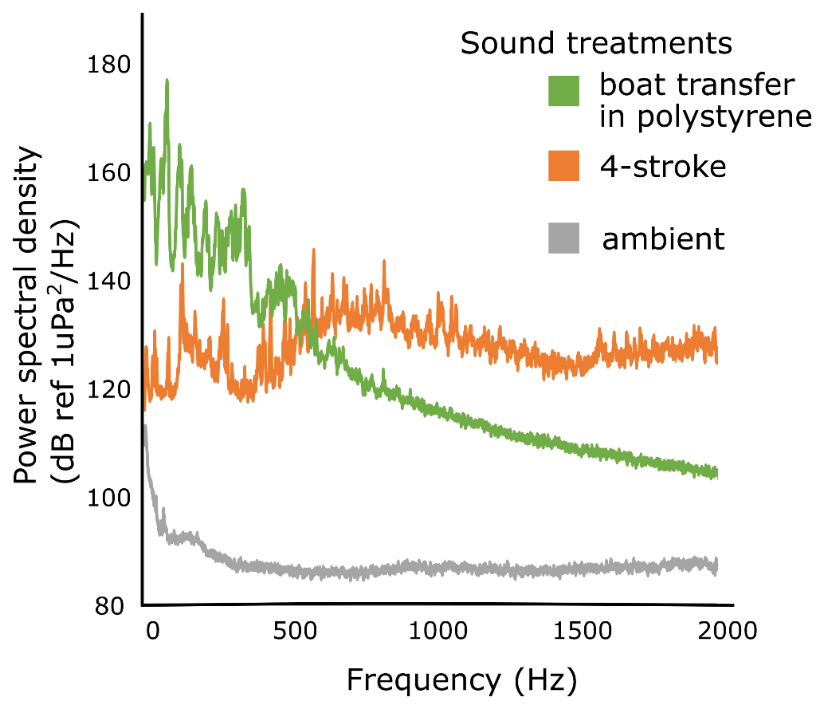


Figure S1. Sound pressure mean (RMS) power spectral density that *Amblyglyphidodon curacao* embryos were exposed to during transport in a polystyrene container on a 4-stroke powered boat to the experiment location, compared to 4-stroke powered boat noise from underwater and underwater ambient noise. The boat was travelling at 20 km/h during transport and 0–35 km/h, 10–200 m from the sound recording device during the underwater recording. Analysis was conducted in paPAM.


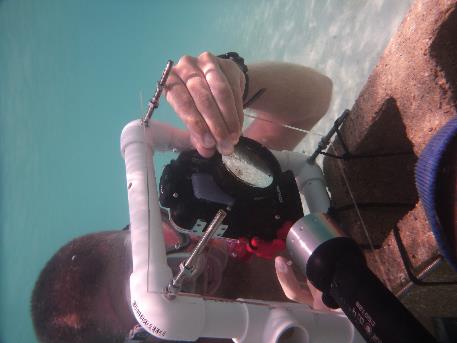


Figure S2. Photograph of heart rate video recording setup used to examine heart rate responses of *A. curacao* embryos to real boat noise.
